# Epidemiological and Virological Characterization of Influenza B Virus Infections

**DOI:** 10.1371/journal.pone.0161195

**Published:** 2016-08-17

**Authors:** Sivan Sharabi, Yaron Drori, Michal Micheli, Nehemya Friedman, Sara Orzitzer, Ravit Bassal, Aharona Glatman-Freedman, Tamar Shohat, Ella Mendelson, Musa Hindiyeh, Michal Mandelboim

**Affiliations:** 1 Central Virology Laboratory, Ministry of Health, Chaim Sheba Medical Center, Ramat-Gan, 52621, Israel; 2 Department of Epidemiology and Preventive Medicine, School of Public Health, Sackler Faculty of Medicine, Tel-Aviv University, Tel-Aviv, Israel; 3 The Israel Center for Disease Control, Israel Ministry of Health, Tel-Hashomer, 52621, Israel; 4 Department of Family and Community Medicine, New York Medical College, Valhalla, New York; Icahn School of Medicine at Mount Sinai, UNITED STATES

## Abstract

While influenza A viruses comprise a heterogeneous group of clinically relevant influenza viruses, influenza B viruses form a more homogeneous cluster, divided mainly into two lineages: Victoria and Yamagata. This divergence has complicated seasonal influenza vaccine design, which traditionally contained two seasonal influenza A virus strains and one influenza B virus strain. We examined the distribution of the two influenza B virus lineages in Israel, between 2011–2014, in hospitalized and in non-hospitalized (community) influenza B virus-infected patients. We showed that influenza B virus infections can lead to hospitalization and demonstrated that during some winter seasons, both influenza B virus lineages circulated simultaneously in Israel. We further show that the influenza B virus Yamagata lineage was dominant, circulating in the county in the last few years of the study period, consistent with the anti-Yamagata influenza B virus antibodies detected in the serum samples of affected individuals residing in Israel in the year 2014. Interestingly, we found that elderly people were particularly vulnerable to Yamagata lineage influenza B virus infections.

## Introduction

Influenza viruses, which are members of the *Orthomyxoviridae* family, are divided into three genera, A, B, and C [1,2]. Influenza A and B viruses are of clinical relevance, since they cause severe respiratory infections in humans and contribute to increased morbidity and mortality globally [3]. While influenza A viruses infect both humans and animals including swine, birds, and horses, influenza B viruses circulate primarily in humans. Several reports have suggested that seals can serve as a possible animal reservoir for influenza B viruses [4]. The clinical symptoms associated with influenza B virus infections are generally similar to those of the influenza A virus [5–9].

Influenza A viruses comprise a large group that can be divided into 16 different HA subtypes (H1–H16) and nine different NA subtypes (N1–N9), based on HA amino acid differences. In contrast, influenza B viruses are more homogenous, and only began to diverge into two main antigenically distinguishable B/Victoria/2/87 and B/Yamagata/16/88 around 1970 [10,11]. HA protein sequences within each of the two lineages are more than 97% identical, with sequence identity in inter-lineage comparisons averaging 88–90% [12,13].

Influenza B viruses Victoria and Yamagata lineages have recently co-circulated in many regions of the world. Although the trivalent seasonal influenza vaccines include one lineage of influenza B virus, evidence suggests that the influenza vaccines can be improved by including both lineages [5]. Here, we report the lineage analysis of influenza B virus infections between 2011 and 2014 among hospitalized Israeli patients and in the general population.

## Materials and Methods

### Patients and samples

Respiratory samples (nasopharyngeal swabs or aspirates) were collected between October 2011 and May 2014, from patients hospitalized at Chaim Sheba Medical Center, Israel, and from patients with influenza-like-illness (ILI) referred to sentinel clinics throughout Israel. In the latter cases, samples were collected from over 20 outpatient clinics, as part of the ICDC government-approved surveillance program for respiratory viruses. The study included 19,714 non-duplicate patients: 15,035 hospitalized patients and 4,679 samples from the community (ICDC surveillance). Of the 4,679 survey samples, 609 were positive for influenza A, 361 for influenza B and 358 for the pandemic H1N1 virus. Out of the 15,035 samples of hospitalized patients, 946 were positive for influenza A, 338 for influenza B and 538 for the pandemic H1N1 virus.

Well characterized serum samples (N = 760) were obtained from samples deposited in 2014 at the Israel national serum bank established by the Israel Center for Disease Control. The serum samples were classified based on and gender and every fifth sample was selected for the analysis. The vaccination history of the samples was unknown.

Only age groups, gender and geographical residency regions in Israel were available to the researchers and patient identity was kept anonymous. Samples from patients suffering from immunological disorders were not included.

### Viral genome extraction and real-time PCR analysis (q-PCR and q-RT-PCR)

Viral genome extraction from 500μl patient respiratory samples, eluted in 55μl elution buffer, was performed with NucliSENS easyMAG (BioMerieux, France). All samples were stored at -70°C until analysis. Hospitalized patient samples were tested by qPCR or qRT-PCR for the presence of the following common human respiratory viruses: adenovirus, human metapneumovirus (hMPV), respiratory syncytial virus (RSV), influenza viruses (A, B, and H1N1pdm) and parainfluenza virus-3 [14]. Sentinel samples from non-hospitalized patient were tested for the presence of influenza A and B viruses and RSV. In the qRT-PCR assay, the influenza B-specific primers amplified part of the HA gene (nucleotide positions 970 to 1139) [15]. The sequences of the influenza virus B-specific forward and reverse primers were as follows: primer INFB-1, 5′-AAATACGGTGGATTAAATAAA AGCAA-3′; primer INFB-2, 5′-CCAGCAATAGCTCCG AAGAAA-3′. The influenza virus B-specific probe was labeled with VIC at the 5′end (VIC-5′-CACCCATAT TGGGCAATTTCCTATGGC-3′-TAMRA).

Reactions were performed in 25μl Ambion Ag-Path Master Mix (Life Technologies, USA) using TaqMan Chemistry on the ABI 7500 instrument.

### Determination of influenza B lineages and phylogenetic analysis

All influenza B-positive samples were subjected to a second round of qRT-PCR, which used one set of primers and two different probes in order to determine the influenza B type (Yamgata and Victoria), as previously described [16].

The influenza B forward and reverse primers were: F432 5′-ACCCTACARAMTTGGAACYTCAGG-3′, and R479 (5′-ACAGCCCAAGCCATTGTTG-3′). The Yamagata probe was MGB437 5′-FAM-AATCCGMTYTTACTGGTAG-MGB-3′, and the Victoria probe was MGB470 (5′-VIC-ATCCGTTTCCATTGGTAA-MGB-3′.

For the phylogenetic analysis, influenza B-positive samples from each lineage were randomly selected and subjected to RT-PCR amplification of the HA gene, using Influenza B forward primer BHA-F1 5' AATATCCACAAAATGAAGGCAATA 3'

And Influenza B reverse primer BHA-R1166 5' ATCATTCCTTCCCATCCTCCTTCT 3'.

The PCR products were then sequenced using ABI PRISM Dye Deoxy Terminator cycle sequencing kit (Applied Biosystems, Foster City, CA). Reaction mixtures were analyzed on Applied Biosystems model 3100 DNA automatic sequencing systems. The Sequencher^®^ 5.0 program (Gencodes Corporation, Ann Arbor, MI) was used to clean and compare the nucleotide sequences.

To infer the evolutionary relationships and the most recent common ancestor (MRCA) for the influenza B virus hemagglutinin sequences, a Bayesian Markov chain Monte Carlo (MCMC) method was applied using a relaxed molecular clock, as implemented in the BEAST program (version 1.7.5). Trees were visualized and edited with the FigTree program (version 1.4.2) included in the BEAST software package [17].

### Hemagglutination inhibition assay

All patient sera were treated for 16 h, with receptor destroying enzyme (RDE) (Sigma C8772), diluted 1:4, prior to heat inactivation (30 min, 56°C) and absorption with erythrocytes to remove non-specific hemagglutination, in accordance with a modified WHO protocol, as recommended by [18]. Serial two-fold dilutions (1:20–1:2560) of sera in 25μl PBS were prepared in V-shaped well plates, and an equal volume of four hemagglutinin (HA) units of viral antigen were added. The mixture was then incubated at room temperature for 1 h. Fifty microliters of 0.5% chicken erythrocytes suspended in PBS were added to the wells, and mixed by shaking the plates on a mechanical vibrator. Agglutination patterns were read after 30 min and the Henmmagglutination Inhibition (HI) titer was defined as the reciprocal of the last dilution of serum that fully inhibited hemagglutination. The cut-off value selected for a positive result was 1:40. The influenza B antigens (B/Brisbane/60/2008- Victoria and B/Massachusetts/2/2012 –Yamagata) were supplied by the WHO.

### Microneutralization assay

Human sera were heat-inactivated, and two fold serial dilutions of 1:20–1:2560 were prepared in immunoassay plates. The diluted sera were mixed with an equal volume of Dulbecco’s modified Eagle’s medium with 2% fetal bovine serum and antibiotic, supplemented with 100 TCID50/50 ml influenza virus (B/Brisbane/60/2008 or B/Massachusetts/2/2012). After 1 h incubation at 37°C, MDCK cells were added to each well. The plates were then incubated for 20 h at 37°C. The supernatant was removed, and monolayers were washed with PBS and fixed in 80% cold acetone for 10 min.

The presence of viral protein was detected by ELISA, as previously described [18–20], using a mouse monoclonal Influenza Type B antibody pool supplied by the WHO. The fixed plates were washed three times with PBS containing 0.05% Tween 20 (wash buffer). The anti-B antibodies, diluted 1/4,000 in PBS containing 1% bovine serum albumin and 0.1% Tween 20 (E diluent), were added to each well, which were then incubated at room temperature for 1 h. The plates were washed four times in wash buffer, and 100μl of horseradish peroxidase-labeled goat anti-mouse immunoglobulin G (IgG) (Dako, USA), diluted 1/2,000 in E diluent, was added to each well. The plates were incubated for 1 h at room temperature and then washed six times with wash buffer. One hundred microliters of freshly prepared substrate (10 mg o-phenylenediamine dihydrochloride per 20 ml 0.05 M phosphate citrate buffer, pH 5.0, containing 0.03% sodium perborate) were added to each well, and the plates were incubated at room temperature for approximately 5 min. The reaction was terminated with an equal volume of 1N sulfuric acid. The absorbance was measured at 490.

### Statistical analysis

The chi-square test was applied to evaluate the differences in percent positivity between the compared groups. A *p* value <0.05 was considered statistically significant. All analyses were performed using SPSS (version 21.0.0. SPSS Inc., Chicago, IL, USA), SAS (SAS 9.1, SAS Institute Inc, Cary, NC, USA) and Excel software.

### Ethical considerations

This was a retrospective study performed on anonymous patient samples that were analyzed for the presence of various common human respiratory viruses, as part of routine clinical testing performed at the Sheba Medical Center in Israel. Additional samples were obtained from patients visiting sentinel clinics spread throughout Israel, as part of community influenza surveillance, conducted in collaboration with the Israel Center for Disease Control (ICDC). No repeat samples were collected from the same patients for this study, therefore, an informed consent was not required. The institutional review board (IRB) of the Sheba Medical Center approved this research (Helsinki Number 9750-12-SMC). Serum samples (N = 760) were obtained from the Israel national serum bank established by the Israel Center for Disease Control. The samples were collected from individuals in 2014. The IRB of the Sheba Medical Center approved this part of the research Helsinki Number 2873-15-SMC).

## Results

### Infections with influenza viruses in Israel between 2011–2014

**Fig 1:**
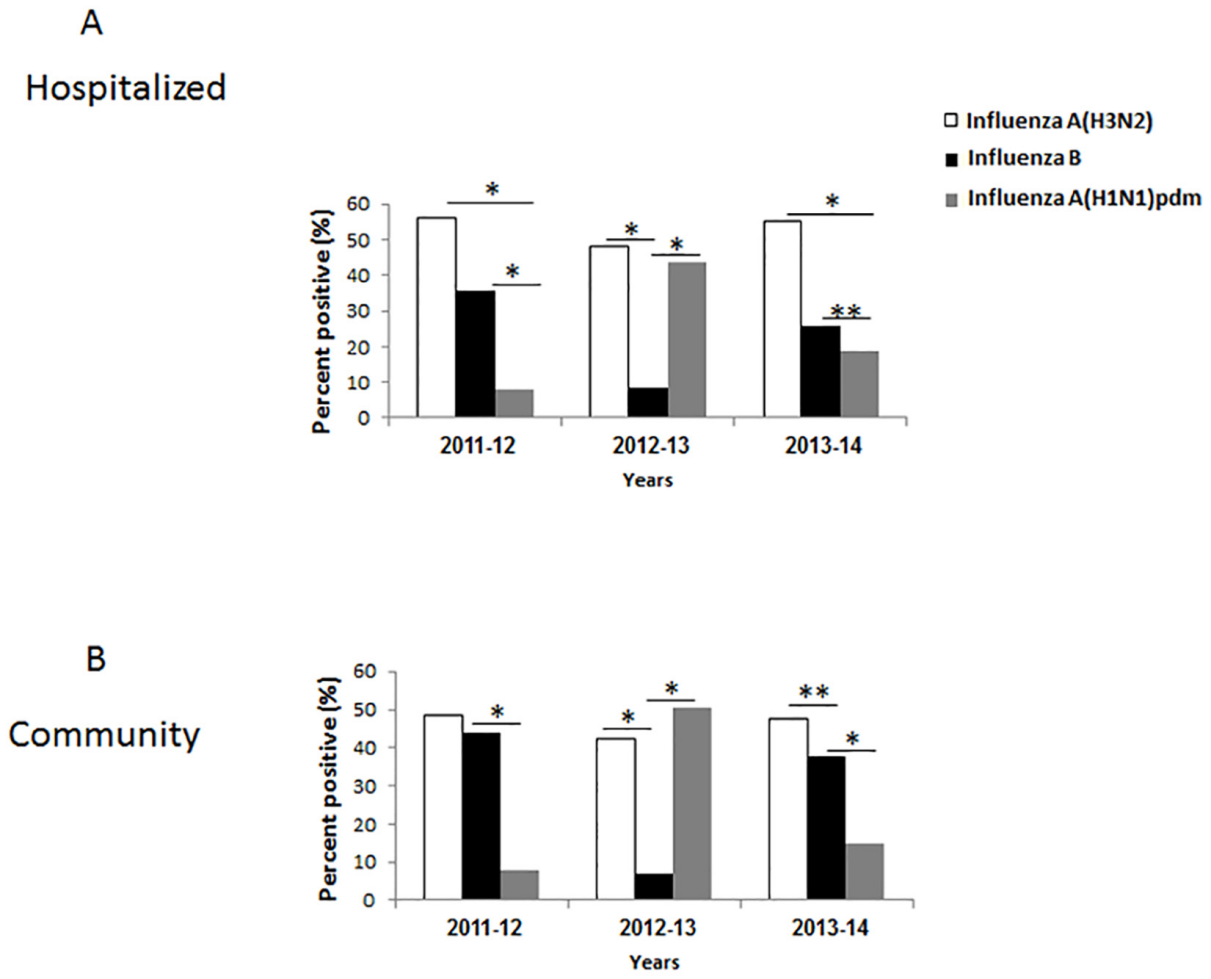
Infections with influenza viruses in Israel between 2011–2014. The percent of hospitalized (A) and community (B) patients infected with influenza A(H3N2), B and A(H1N1)pdm viruses between 2011–2014. *p<0.05.

The distribution of influenza A and influenza B infections between 2011–2014 in patients hospitalized in the Sheba hospital and in the community (ICDC surveillance), is presented in Fig 1A and 1B. Altogether, we tested 15,035 hospitalized patients and 4679 community patients. The infection trend was similar between hospitalized and community patients. In 2011–2012, most hospitalized patients had influenza A(H3N2), fewer patients were infected with influenza B and even fewer were infected with the H1N1pdm virus (Fig 1A). A similar distribution was seen in the community, although the incidence of influenza B infections was closer to that of influenza A (Fig 1B). In 2012–2013, the number of patients infected with influenza A(H3N2) and H1N1pdm was similar in the hospital versus community samples, and fewer hospitalized and community patients were infected with influenza B virus (Fig 1A and 1B). In 2013–2014, most patients were hospitalized due to influenza A(H3N2) infection, while a significantly lower percentage of patients were hospitalized due to influenza B or to H1N1pdm virus infection (Fig 1A). Similarly, in the community there were more influenza A(H3N2) infections than influenza B infections and even fewer H1N1pdm infections (Fig 1B).

### Influenza B virus detection and distribution in Israel between 2011–2014

**Fig 2:**
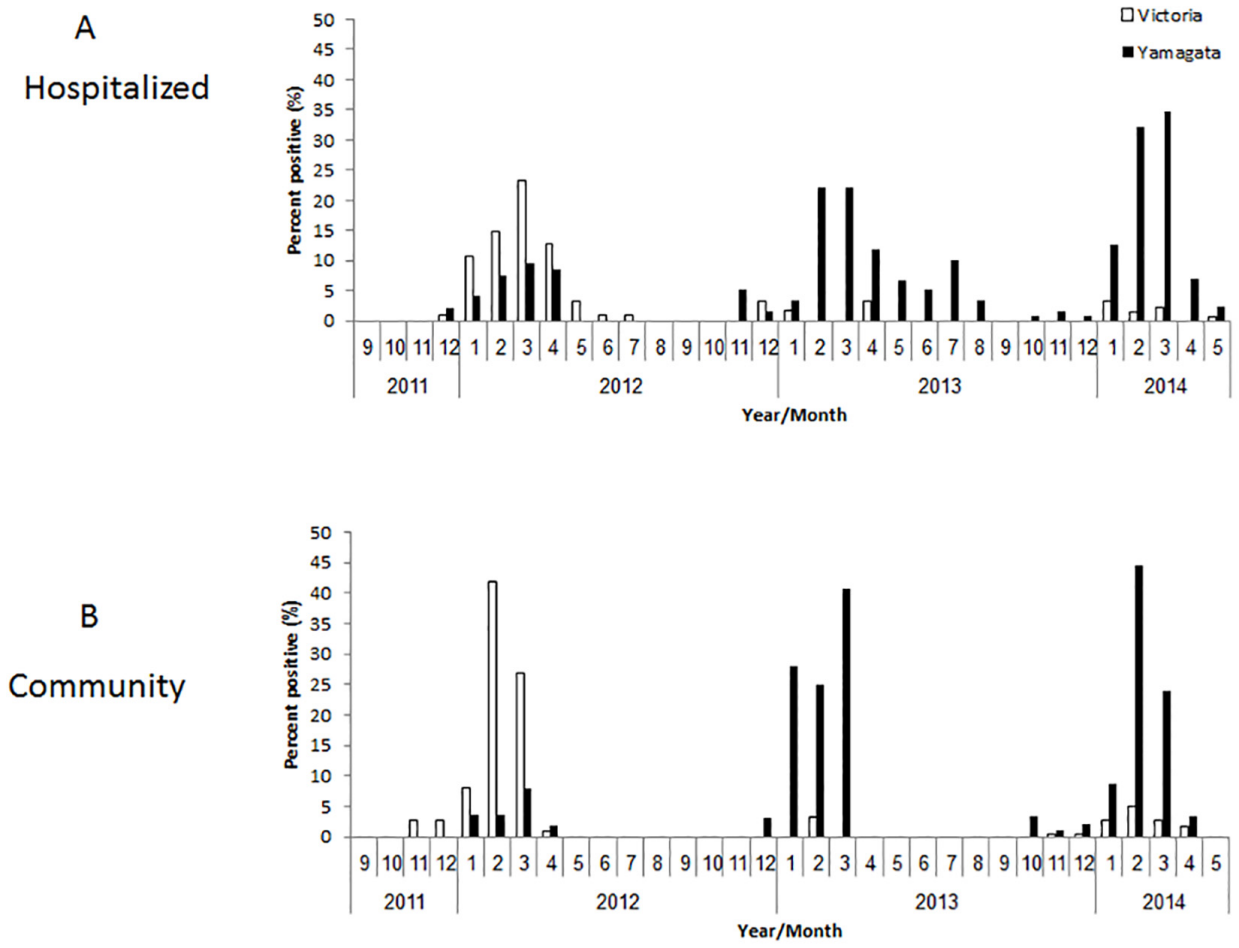
Infections with influenza B viruses in Israel between 2011–2014. The monthly percent distribution of hospitalized (A) and community (B) patients positive for infection of influenza B viruses: Victoria and Yamagata between 2011–2014.

**Fig 3:**
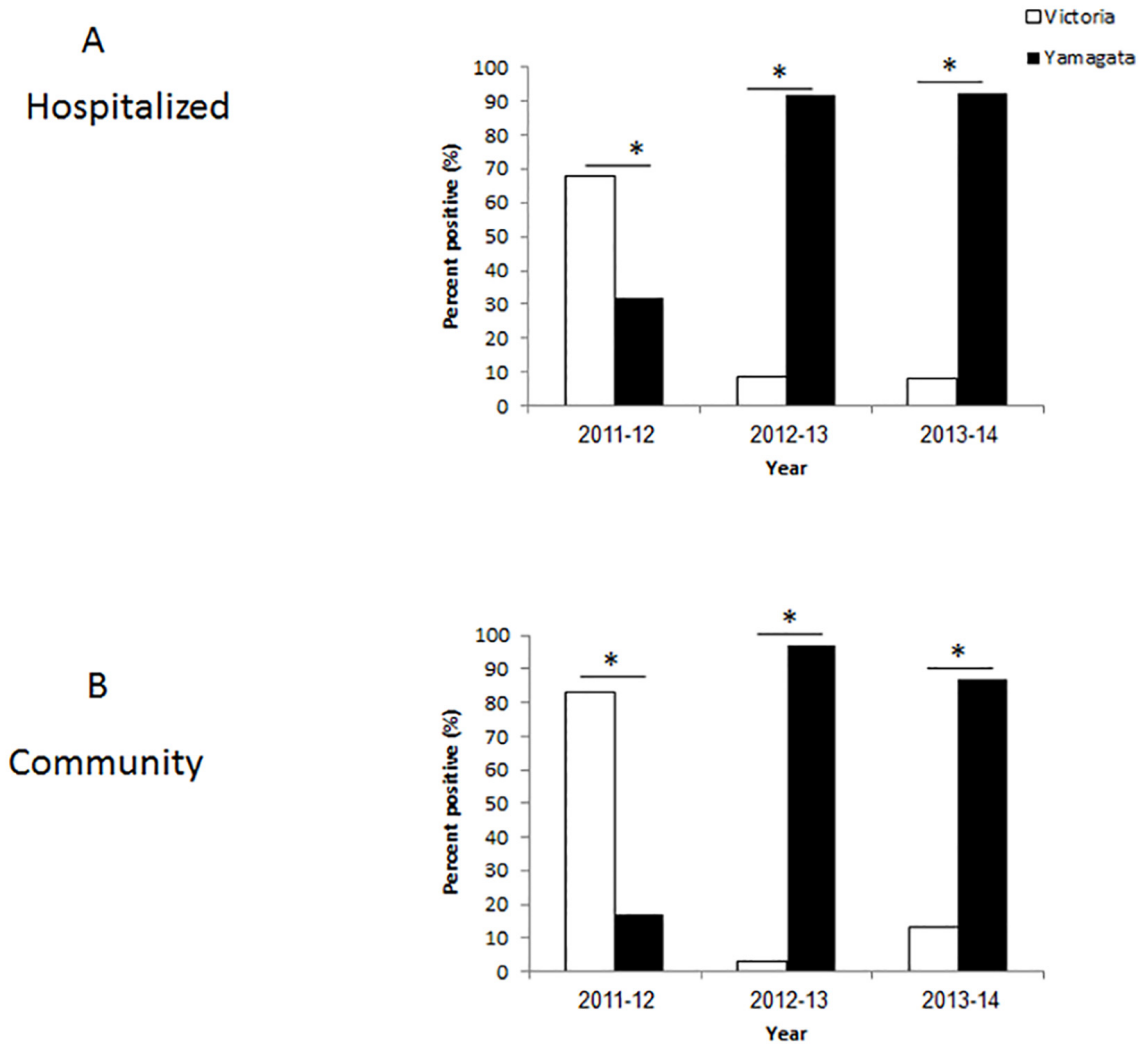
Temporal of influenza B infection. The percentages of hospitalized (A) and community (B) patients, during the winter seasons of 2011–2014. *p<0.05.

The relatively high percentages of influenza B infections prompted us to determine the lineages (Victoria or Yamagata) that infected the hospitalized patients and those that circulated in the community. In 2011–2012, most of the hospitalized and community patients were infected with the Victoria lineage (Fig 2). The vaccine administered that year indeed included the influenza B Victoria virus (Table 1). In 2012–2013, almost all hospitalized and community patients were infected with the Yamagata lineage, (Fig 2A and 2B). The vaccine that year included the Yamagata virus (Table 1). In 2013–2014, most infections were associated again with the Yamagata virus (Fig 2A and 2B), which had been included in the annual vaccine (Table 1). Examination of the temporal distribution of influenza B infections throughout the year, demonstrated a peak of infections, in both hospitalized and community patients, was between January and March, in each of the analyzed years (Fig 2). No significant differences were observed between the two sample sets (hospitalized and community patients, Fig 3).

**Table 1:** Viruses included in the vaccine.

	Influenza A(H1N1)pdm09	Influenza A(H3N2)	Influenza B—Lineage
**2011–12**	A/California/7/2009	A/Perth/16/2009	B/Brisbane/60/2008- Victoria
**2012–13**	A/California/7/2009	/Victoria/361/2011	B/Wisconsin/1/2010—Yamagata
**2013–14**	A/California/7/2009	A/Texas/50/2012	B/Massachusetts/2/2012—Yamagata

### Age and gender distribution

**Fig 4:**
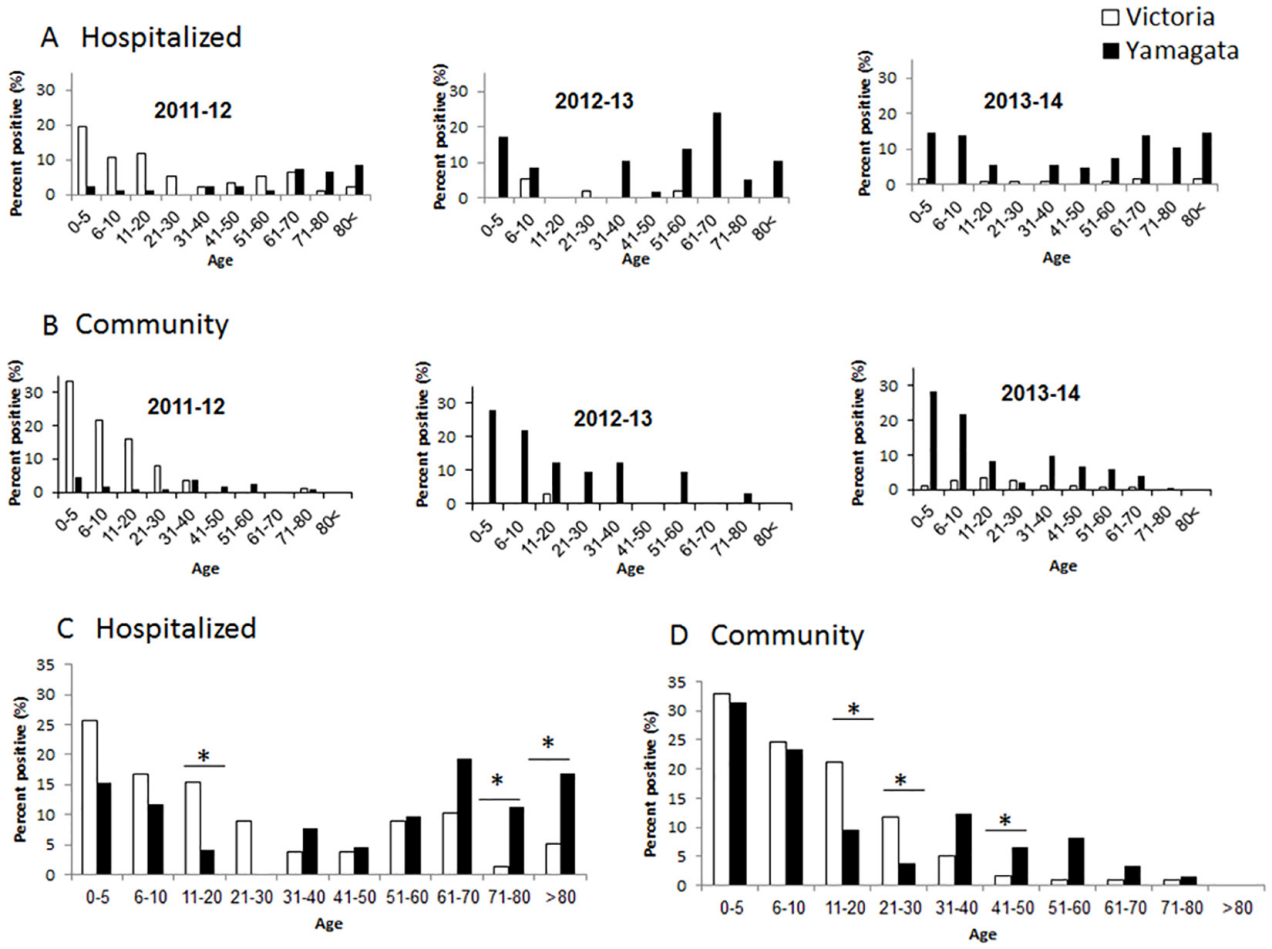
Age distribution of the influenza B-infected patients. The yearly age distribution of hospitalized (A) and community (B) patients positive for infection of influenza B viruses: Victoria and Yamagata between 2011–2014. Summary of the ages over the years in shown in (C-D).

**Fig 5:**
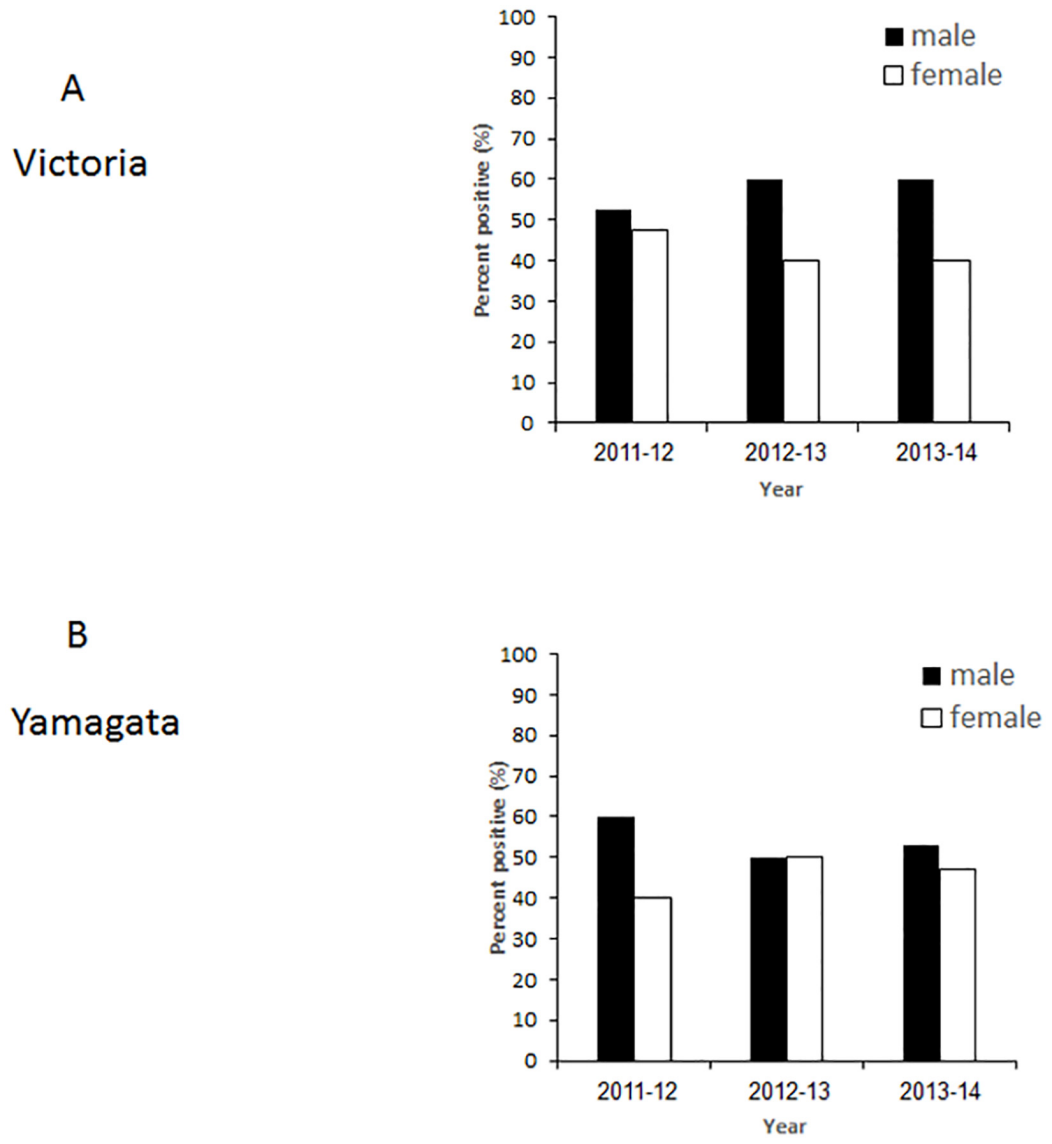
Gender distribution in hospitalized patients. The gender distribution of hospitalized Victoria-infected (A) and Yamagata-infected (B) patients between 2011–2014.

Analyzing the age distribution of patients infected with influenza B during 2011–2014, a dramatic age-associated difference between B Victoria and B Yamagata incidence was noticed. Most of the Victoria virus-infected patients (in 2011–12) were between 0–30 years of age, both in the hospitalized patients and in the community (Fig 4A–4D). In contrast, from 2012–2014, the dominant B virus was Yamanaka, which infected patients of all ages, including the elderly population (Fig 4A–4D). In general, throughout the study period, more men were infected (Fig 5). In 2012–13, the frequency of infection among women and men was similar (Fig 5), however, in that year, only a small number of influenza B infections was available (Fig 1). Information pretending to gender in the infected community patients was not available to us.

### Antibody response to influenza B

**Fig 6:**
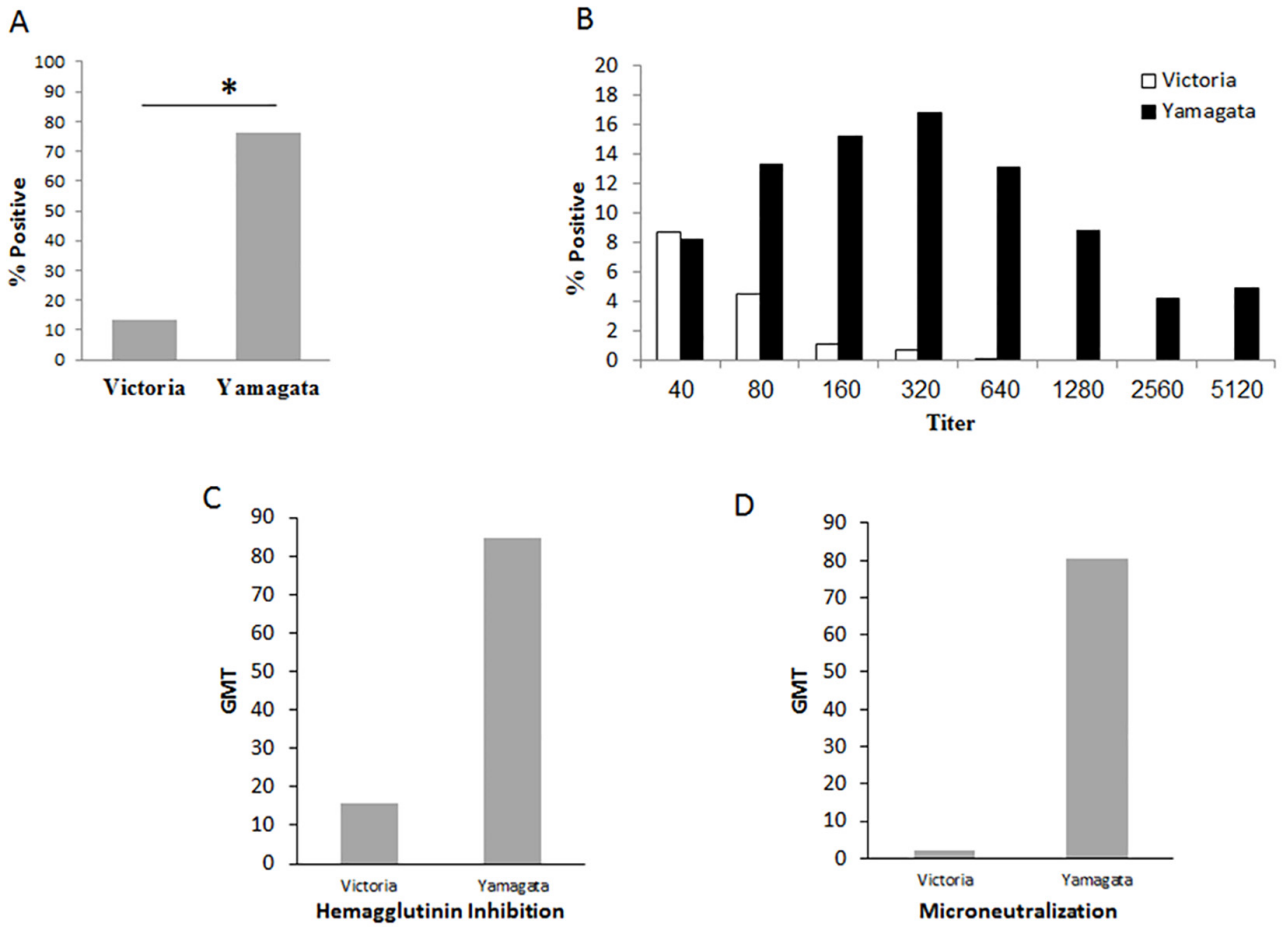
Antibodies against Yamagata were dominant in 2014. Sera were collected from randomly selected individuals and the presence of antibodies against Victoria and Yamagata was detected as described in materials in methods. Summary of positive samples is shown in (A). Shown in (B) is the virus titers and in (C) Geometric Mean Titers (GMT) antibody neutralization of the Israeli circulating influenza B. (D) Microneutralization assays.

Upon analysis of the distribution of antibodies to Influenza B-Victoria and Yamagata in the population, in 2014, around 80% of the infected individuals presented antibodies to Yamagata type and around 10% to the Victoria type (Fig 6A). Antibody titers were higher against Yamagata (Fig 6B summarized in Fig 6C). Microneutralization assays were then performed to test the potency of the antibodies. As can be seen in Fig 6D, the anti-Yamagata antibodies were more efficient in neutralizing the infection, as compared to the anti-Victoria antibodies.

### Phylogenetic analysis

**Fig 7:**
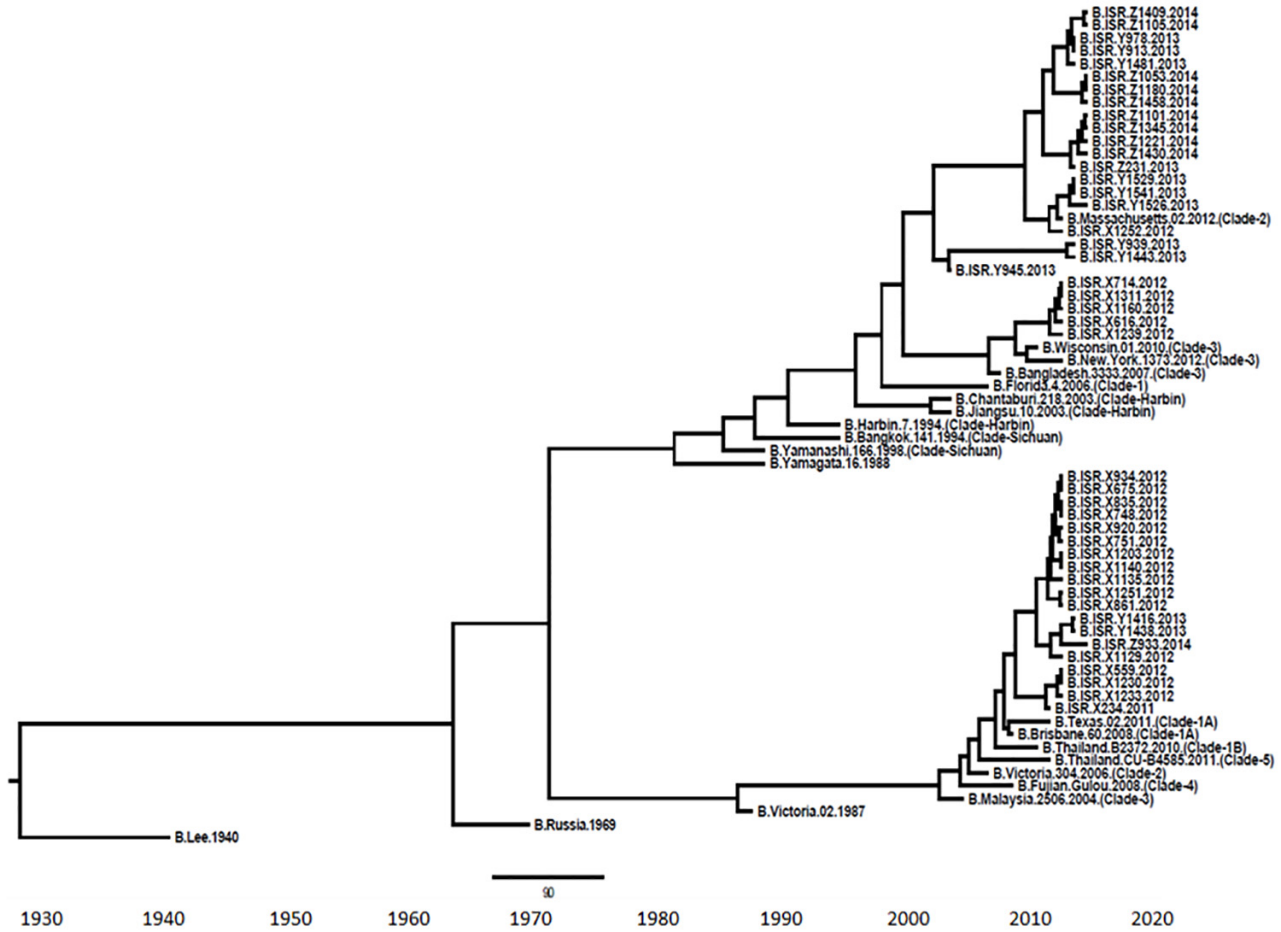
Phylogenetic tree. Bayesian maximum-clade-credibility time-scaled phylogenetic tree (BEAST) generated using 74 partially sequenced influenza B HA gene (476 bp), obtained from patient samples collected between 2012 and 2014 in Israel.

To analyze the specific clades of influenza B viruses circulating in the country during the study period, we performed phylogenetic analysis on 476 bases of the HA gene. The analysis revealed that all Victoria viruses belonged to clade 1A (Fig 7). With respect to the Yamagata viruses, in 2011–2012, clade 2 and 3 were observed, while later on, only clade 2 circulated in Israel (Fig 7). The two lineages diverged in the early 1970’s (Fig 7).

## Discussion

The divergence of influenza B viruses into two lineages, with no antibody cross-protection, has hindered production of effective seasonal influenza vaccines, which have traditionally contained the circulating influenza A strains, H1N1pdm and A(H3N2), and only a single influenza B strain.

During the past decade, selection of the correct influenza B component for the trivalent influenza vaccine, has proven particularly challenging, and lineage-level mismatches between the vaccine and circulating strains of B viruses have occurred in approximately 50% of the seasons [5]. Indeed, we show here, that in Israel, both influenza B clades circulated between 2011–2014, in different proportions. However, we also observed that in each of the three analyzed years, the dominant influenza B lineage in samples collected from infected individuals, was the same as that included in the vaccine. Thus, at least in the tested period, the vaccine included the correct circulating B virus lineage.

The level of cross-protection between the two B lineages is not well known, but it is assumed to be low [21–23]. Few reports have shown that the Influenza B virus Yamagata lineage may induce cross-lineage antibody response for the Victoria lineage, but the opposite is not as efficient [24–26]. In this study, we demonstrated that while the younger population (0–30 years of age) had evidence of both Victoria and Yamagata infection, individuals between 11–30 years of age, were infected mostly by the Victoria virus and individuals >31 years of age were mostly infected with the Yamagata virus. These findings are not consistent with earlier reports that showed dominance of the influenza B Victoria lineage over the Influenza B Yamagata lineage in the young population [27,28]. Elderly individuals tend to be vaccinated against influenza and indeed, in the study period, 60.4% of the individuals above the age of 65 were immunized with the influenza vaccine as compared to 17% in the general population. The dramatic age-associated difference between the incidence of Victoria and Yamagata lineages observed in the 2011–12 season may be very well explained by the fact that the elderly population had been vaccinated against Victoria-like, and hence the only severe cases found in that cohort (those requiring hospitalization) were caused by Yamagata-like strains not covered by the vaccine formulation. This interpretation concurs with available data regarding cross-protection between lineages.
